# Using a mobile nurse mentoring and training program to address a health workforce capacity crisis in Bihar, India: Impact on essential intrapartum and newborn care practices

**DOI:** 10.7189/jogh.10.021009

**Published:** 2020-12

**Authors:** Andreea A Creanga, Safia Jiwani, Aritra Das, Tanmay Mahapatra, Sunil Sonthalia, Aboli Gore, Sunil Kaul, Sridhar Srikantiah, Christine Galavotti, Hemant Shah

**Affiliations:** 1 Department of International Health, Johns Hopkins Bloomberg School of Public Health, Baltimore, Maryland, USA; 2 Department of Gynecology and Obstetrics, Johns Hopkins School of Medicine, Baltimore, Maryland, USA; 3 CARE India Solutions for Sustainable Development, Patna, Bihar, India; 4 CARE USA, Atlanta, Georgia, USA

## Abstract

**Background:**

To address a health workforce capacity crisis, in coordination with the Government of Bihar, CARE India implemented an on-the-job, on-site nurse mentoring and training intervention named – *Apatkalin Matritva evam Navjat Tatparta* (AMANAT, translated Emergency Maternal and Neonatal Care Preparedness) – in public facilities in Bihar. AMANAT was rolled-out in a phased manner to provide hands-on training and mentoring for nurses and doctors offering emergency obstetric and newborn care (EmONC) services. This study examines the impact of the AMANAT intervention on nurse-mentees’ competency to provide such services in Bihar, India during 2015-2017.

**Methods:**

We used data from three AMANAT implementation phases, each covering 80 public facilities offering basic EmONC services. Before and after the intervention, CARE India administered knowledge assessments to nurse-mentees; ascertained infection control practices at the facility level; and used direct observation of deliveries to assess nurse-mentees’ practices. We examined changes in nurse-mentees’ knowledge scores using χ^2^ tests for proportions and *t* tests for means; and estimated proportions and corresponding 95% confidence intervals for routine performance of infection control measures, essential intrapartum and newborn services. We fitted linear regression models to explore the impact of the intervention on nurse-mentees’ knowledge and practices after adjusting for potential confounders.

**Results:**

On average, nurse-mentees answered correctly 38% of questions at baseline and 68% of questions at endline (*P* < 0.001). All nine infection control measures assessed were significantly more prevalent at endline (range 28.8%-86.8%) than baseline. We documented statistically significant improvements in 18 of 22 intrapartum and 9 of 13 newborn care practices (*P* < 0.05). After controlling for potential confounders, we found that the AMANAT intervention led to significant improvements in nurse-mentees’ knowledge (30.1%), facility-level infection control (30.8%), intrapartum (29.4%) and newborn management (24.2%) practices (all *P* < 0.05). Endline scores ranged between 56.8% and 72.8% of maximum scores for all outcomes.

**Conclusion:**

The AMANAT intervention had significant results in a health workforce capacity crisis situation, when a large number of auxiliary nurse-midwives were expected to provide services for which they lacked the necessary skills. Gaps in intrapartum and newborn care knowledge and practice still exist in Bihar and should be addressed through future mentoring and training interventions.

**Study registration:**

ClinicalTrials.gov number NCT02726230.

At the end of 2010, the public health sector in Bihar, India was facing an increasing demand for institutional births [1,2] as a result of the Government of India’s 2005 *Janani Suraksha Yojana* conditional cash transfer program [3]. To address this higher demand and the health workforce shortage in the state, several thousand auxiliary nurse-midwives (ANMs) had been placed in public facilities, mostly primary health centres (PHCs), to offer emergency obstetric and newborn care (EmONC) services [1]. Compared to staff nurses who complete high school and obtain General Nursing and Midwifery (GNM) diplomas after 3 years of nursing school, ANMs complete high school and possess a 2-year diploma upon training to become multipurpose community health workers and to offer mainly outreach services [4]. Importantly, while GNMs are expected to be skilled to manage both normal and complicated labor and delivery, ANMs’ training curriculum provides some theoretical knowledge, but significantly less practical delivery experience [5]. Moreover, ANMs selected to offer services in public facilities throughout Bihar were either identified from lists of recent school graduates or had experience conducting mainly outreach work and most received no refresher training. The Skilled Birth Attendant training program that was being implemented in the state at that time was inadequate in ensuring a minimum level of quality of intrapartum care [5]. In the cultural context of Bihar, the few available male doctors would not enter the labour room; female doctors were even fewer. This situation had created a serious health workforce capacity crisis in Bihar in the face of a massive and rapid rise in institutional deliveries in public facilities.

In coordination with the Government of Bihar (GoB), in 2011, CARE India and other non-governmental organisation partners embarked on providing support to the GoB in implementing the *Ananya* program to improve maternal and neonatal outcomes and quality of care in the state [1]. The program was designed to, inter alia, support the strengthening of EmONC services in public sector facilities. Given the large number of providers, especially ANMs, expected to offer such services without adequate training and skills, and the challenges associated with organising off-site skill training, the solution CARE India identified was on-the-job, on-site mentoring and training, using highly qualified nurses as mobile nurse mentors.

The intervention was implemented jointly by CARE India and the GoB, first in 80 public facilities offering basic EmONC (BEmONC) services in eight pilot districts in Bihar during 2012-2014. This preceded the statewide scale-up of the intervention named *Apatkalin Matritva evam Navjat Tatparta* (AMANAT, translated Emergency Maternal and Neonatal Care Preparedness) in 2015 [1,6], following the expansion of the CARE India program interventions statewide through the constitution of a Bihar Technical Support Program (BTSP) [1]. In facilities expected to provide BEmONC services, AMANAT-B (*Buniyadi*, or basic) mentoring and training focused on nursing staff’s conduct of normal deliveries and the identification, stabilisation, and referral of maternal and neonatal complications. AMANAT-B was rolled-out in a phased manner, with similar program content across phases. A doctors’ mentoring program component was combined with nurse mentoring in district hospitals to offer a comprehensive EmONC (CEmONC) mentoring intervention during 2014-15 – it was designed to focus on emergency response and case management of maternal and neonatal complications. After being implemented in five district hospitals in the initial eight pilot districts, the CEmONC mentoring intervention was expanded as AMANAT-V (*Vyapak*, or comprehensive) statewide. However, given the insufficient number of qualified doctor-mentors willing to engage in doctor mentoring in Bihar, this component of the AMANAT program could not be implemented with the envisaged intensity. It covered only 23 district hospitals, with the nurse mentoring component being more intensive than the doctor mentoring component [6].

Results from the eight-district phase that preceded the AMANAT program showed significant improvements in essential maternal and newborn practices performed by staff nurses [7,8]. Of note, these improvements were ascertained using direct observation of care, considered the “gold standard” approach for evaluating quality of care because it identifies points in the care process where quality improvements are needed [9]. The AMANAT intervention also benefitted from a concurrent evaluation with a quasi-experimental, pre-post design that employed knowledge surveys and direct observation of care. This evaluation provides a unique opportunity to consolidate the evidence from the eight-district pilot regarding the impact of a mobile on-the-job, on-site nurse mentoring and training intervention in the context of a health workforce capacity crisis in Bihar. Specifically, this article examines the impact of the AMANAT-B (hereafter AMANAT) intervention on nurse-mentees’ competency to provide essential intrapartum and newborn care services in the state during 2015-2017.

## METHODS

Eighty public BEmONC facilities were purposively selected to participate in each of four phases of the AMANAT program during 2015-2017. In each phase, selection of facilities was based on assessments of facility readiness for the AMANAT program, including 1) a minimum of six nurse-mentees available for training in each facility, 2) an average load of ≥100 deliveries each month, 3) basic readiness in terms of infrastructure and equipment, and 4) willingness of the facility management to participate. Decisions regarding facilities’ inclusion into various AMANAT phases also considered the proximity of intervention-ready facilities. Nurse-mentors with BSc degrees in nursing and at least two years of experience providing BEmONC services were recruited from across India in partnership with the Christian Medical Association of India through a rigorous interview process. They were familiarised with the training modules by master trainers holding master’s degrees in nursing. Pairs of nurse-mentors rotated weekly through four facilities over a period of 6-8 months. They used structured learning sessions to cover a range of topics (ie, basic nursing procedures, infection prevention, essential obstetric and newborn practices, management of complications such as postpartum hemorrhage or pre-eclampsia, teamwork and communication, documentation and reporting), as well as bedside mentoring related to the management of normal and complicated labor and delivery. The approach was learning-by-doing to the extent possible, utilising opportunities presented by the women admitted daily for delivery in each facility and enabling supervised improvements such as organising labor rooms for greater efficiency, ensuring correct storage and use of drugs and sterilisation of equipment, correct and complete clinical documentation, and periodic clinical case reviews. Despite about 50 days of intermittent exposure to mentoring, there was only limited exposure of individual mentees to the clinical management of even common complications such as postpartum hemorrhage and birth asphyxia. To make up for this gap, high-fidelity simulation training using inexpensive props, video recordings, and debriefing techniques designed by Pronto International were embedded into the AMANAT intervention and used for team-training facilitated by experts from the University of California San Francisco and University of Utah — these were focused primarily on the identification and management of intrapartum complications [6,10-12], and are described in detailed in another article in this series [11]. Highly utilised were *MamaNatalie* and *NeoNatalie*, simulation models used to demonstrate obstetric and neonatal practices. Mentoring and training were conducted in groups, in the local Hindi dialect. Nurse-mentors worked with the nurse-mentees to observe, provide instruction, and demonstrate through co-management of cases. In addition, they were available by phone during non-mentoring weeks (ie, between the week-long mentoring visits) to offer patient management guidance and enable nurses to practice per protocol.

For this analysis, we use data from the last three of four implementation phases of the AMANAT intervention, each covering blocks of 80 public facilities offering BEmONC services between September 2015 and January 2017; owing to their formative nature, baseline data from the first implementation phase were judged not suitable for pre-post intervention comparisons. Evaluation data were collected the week before the intervention (baseline) and approximately a month after completion of the intervention (endline) in all facilities where the intervention was implemented. In each facility, all nurse-mentees posted in the labour room were administered knowledge tests with 11 common questions and 19 content-similar questions at baseline and endline. General infection control measures in place in each facility were captured on separate checklist-based tools at baseline and endline. To assess maternal intrapartum and newborn clinical practices, CARE employed direct observation of deliveries by nurse-mentors, using a pre-formatted, pilot-tested tool to record the execution of key care steps and procedures. For each phase of 80 facilities, nurse mentors assigned to mentor the facilities conducted baseline observations before beginning the training over the period of one week. After the end of the mentoring period of 6-8 months, a different set of nurse mentors conducted endline observations for that phase over the period of one week. Only normal deliveries conducted by nurse mentees on Monday to Friday, 8 am-5 pm during the week when the evaluation team was present in the health facility were selected for observation. Complicated deliveries were not included in either pre- or post-assessments.

Outcomes of interest for our analysis were: 1) nurse-mentees’ knowledge score measured as the sum of correct responses to the 30 knowledge questions; 2) facility infection control score measured as the sum of routinely (ie, throughout the week of assessment) performed infection control measures out of nine measures assessed in each facility; 3) intrapartum clinical practices measured as the sum of correctly performed practices out of 22 practices assessed for all deliveries observed; and 4) newborn clinical practices measured as the sum of correctly performed practices out of 13 essential newborn practices assessed for all newborns observed. Items assessed represent essential elements of intrapartum and newborn care and were scored 1 if performed and 0 if not performed as per accepted clinical guidelines in India [13]. Given that only essential practices performed during normal labour and delivery were assessed, score items were weighted equally.

**Figure 1:**
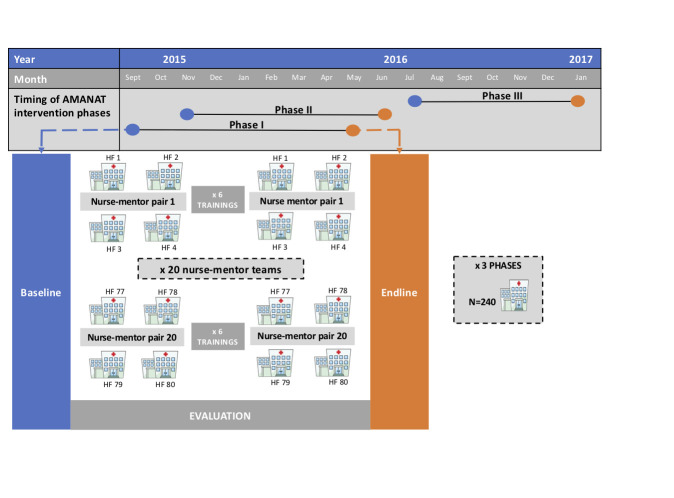
Implementation of the AMANAT intervention between September 2015 and January 2017. AMANAT – *Apatkalin Matritvaevam Navjat Tatparta*, translated emergency obstetrical and neonatal readiness. Blue dots indicate start of data collection; orange dots indicate end of data collection for each phase; each of the three phases covered 80 facilities offering basic emergency obstetric care. The evaluation was on-going, with baseline data collection occurring before the intervention and endline data collection occurring about one month after the end of the intervention.

Across the three phases with data analysed here, the AMANAT intervention was implemented in 240 public facilities. Due to fieldwork problems (eg, travel delays, absenteeism and overlapping evaluation timing for nurse-mentors coming from outside Bihar; **Figure 1**), only 206 facilities had available data for all outcomes of interest at both baseline and endline. Because randomisation of deliveries and newborns observed was not feasible, additional information was collected during the evaluation and used in analyses. The facility type was categorised as PHC or higher-level; 2014 facility delivery volumes were obtained from the Health Management Information System (HMIS) [14] and used in analyses as a continuous variable. A measure of both available and functional facility equipment was created based on assessment of 10 types of equipment needed to perform essential intrapartum and newborn procedures (blood pressure apparatus, fetal doppler, baby weighing machine, radiant warmer, suction machine, neonatal ambu bag, neonatal mask size 0, neonatal mask size 1, oxygen concentrator, oxygen cylinder). For each equipment that was available and functional at the time of assessment, the facility received a score of 1; the equipment score is a summed score of available and functional equipment ranging between 0 and 10. Nurse-mentees’ experience was categorized as <5, 5-9, and ≥10 years. For all observed deliveries, observers captured patients’ age and parity, the time in relation to delivery when the observation started, the time when the woman was taken to the delivery room (before or after 2pm) as a proxy for nurses’ shifts, and the type of other providers present in the room at the time of delivery (doctor/staff nurse vs ANM) to account for the potential heightened attention and higher care quality offered by nurse-mentees when higher-qualified providers were present.

Nurse-mentee and observed delivery characteristics were compared between baseline and endline using χ^2^ tests for proportions, *t* tests for means, and Wilcoxon tests for medians. Subsequently, baseline-endline changes in nurse-mentees’ knowledge were assessed as proportions of mentees who answered knowledge questions correctly, overall and by content (ie, biomedical waste, maternal intrapartum care, newborn care). The statistical significance of baseline-endline knowledge differences was tested using χ^2^ tests for proportions and *t* tests for means. We estimated proportions and corresponding 95% confidence intervals for routine performance of nine infection control measures at the facility level, and of 22 intrapartum and 13 newborn practices at the observed delivery and newborn levels. Lastly, we fitted four linear regression models to explore the impact of the AMANAT intervention (endline vs baseline) and, separately, nurses’ knowledge, infection control, intrapartum, and newborn care practices. To ease the interpretation of results, outcome scores were linearly transformed to a 0-100 scale for use in regression analyses. All models were adjusted for type of facility, 2014 delivery volume, equipment score, AMANAT implementation phase, and clustering at the nurse-mentor pair level given that the same pair trained nursing staff in multiple facilities. The model fitted for nurse-mentee knowledge was also adjusted for nurse-mentee’s experience measured in years. Models fitted for intrapartum and newborn practice performance were also adjusted for nurse-mentee’s experience, observed patient’s parity, the time when the delivery observation started, the time when the woman was taken to the delivery room (before or after 2pm), and the highest qualification of the other providers assisting the delivery.

This study is part of the *Ananya* Bihar program and is registered at ClinicalTrials.gov number NCT02726230. The study was approved by the Institutional Committee for Ethics and Review of Health Management Research Office of the Indian Institute of Health Management Research in Jaipur, India. Written informed consent or left thumb impression was obtained from each participant before the interview or care observation. We used STATA 15 (StataCorp, College Station, TX, USA) to conduct the analyses.

## RESULTS

The 240 AMANAT facilities in the three implementation phases analysed here represent 36 of 38 districts and 48.5% of facilities offering BEmONC services in Bihar (**Table 1**). Our analytic sample includes data collected in 206 (85.8%) of these 240 facilities, four-fifths of which were PHCs. The median number of deliveries performed in these facilities in 2014 was 2124. Overall, 1892 and 1822 nurse-mentees completed knowledge assessments at baseline and endline, respectively. More deliveries were observed at baseline than endline (mean 3.7 vs 3.0 deliveries per facility). Patient observations started closer to their arrival/admission time at endline than baseline, and considerably more deliveries were observed in the morning than the afternoon shift at both baseline and endline.

**Table 1:** Characteristics of facilities, nurse-mentees evaluated, and deliveries observed during the implementation of the AMANAT intervention

Characteristics	Baseline	Endline	*P*-value	
**N (%)**	
Intervention coverage*				
Number of districts of total districts in Bihar	36 of 38 (94.7%)		--	
Number of 3-phase AMANAT-exposed facilities of total BEmONC facilities in Bihar	240 of 495 (48.5%)			
Number of 3-phase AMANAT-exposed facilities with baseline and endline data assessing all knowledge, infection control, and direct observation of deliveries	206 of 240 (85.8%)			
***Facility characteristics (N = 206)***				
Facility level^†^				
PHC	160 (77.7)		--	
Higher level	46 (23.3)			
2014 facility delivery volume^‡^				
-Mean (SD)	2296 (1,236)		--	
-Median (interquartile range, IQR)	2124 (1,416-3,014)			
Facility score of available and functional equipment ^§^				
Mean (std dev)	7.2 (1.9)	8.2 (1.3)	<0.001	
Median (IQR)	7 (6-8)	8 (8-9)	<0.001	
***Evaluated nurse-mentee characteristics***				
Total number nurse-mentees	1,892	1,822	–	
Mean (std dev)	9.2 (3.7)	8.8 (3.4)	0.735	
Median (IQR)	8 (7-9)	8 (7-9)	0.869	
Mentees’ nursing experience (years):				
<5	327 (17.3)	318 (17.6)	0.827	
5-9	654 (34.6)	645 (35.7)		
≥10	876 (46.3)	845 (46.7)		
Missing	35 (1.9)	14 (0.8)		
***Observed delivery characteristics***				
Number of deliveries directly observed	763	628	–	
-Mean (std dev) per facility	3.7	3.0	<0.001	
-Median (IQR) across facilities	3 (1-14)	2 (1-9)	<0.001	
Age of the women whose deliveries were observed (years):				
<20	45 (5.9)	23 (3.6)	0.372	
20-24	388 (50.8)	320 (50.9)		
25-29	276 (36.2)	212 (33.9)		
30+	54 (7.1)	73 (11.6)		
Parity of the women whose deliveries were observed:				
0	202 (26.5)	192 (30.5)	0.412	
1	238 (31.2)	191 (30.4)		
2-3	259 (33.9)	197 (31.5)		
4+	64 (8.4)	48 (7.6)		
Time when observation started:				
Arrival/admission	197 (25.9)	222 (35.4)	<0.001	
In maternity ward before delivery	206 (27.0)	172 (27.4)		
Upon move to labor room	185 (24.1)	107 (16.9)		
In labor room before delivery	175 (23.0)	127 (20.2)		
Time when woman taken to delivery room:				
-Before 2 pm	547 (71.7)	440 (70.0)	0.012	
-After 2 pm	178 (23.4)	167 (26.5)		
-Not recorded	38 (5.0)	21 (3.3)		
Highest qualification level of other providers also assisting the delivery^||^				
-Doctor/Grade A nurse	91 (12.0)	72 (11.5)		0.786
-Auxiliary nurse midwife	672 (88.0)	556 (88.5)			

AMANAT – *Apatkalin Matritvaevam Navjat Tatparta*, translated Emergency Maternal and Neonatal Care Preparedness, SD – standard deviation

*Between September 2015 and January 2017.

† Higher-level facilities include community health centers, first referral units, or sub-divisional hospitals.

‡Facility volume data from Health Management Informations System 2014 data available at: https://nrhm-mis.nic.in/hmisreports/frmstandard_reports.aspx.

§Equipment score with 10 items necessary for providing essential intrapartum and newborn care.

||Only deliveries conducted by nurses were observed, but other providers may be assisting with deliveries; BEmOC, basic emergency obstetrics care; --, not available.

### Nurse-mentee knowledge

**Figure 2:**
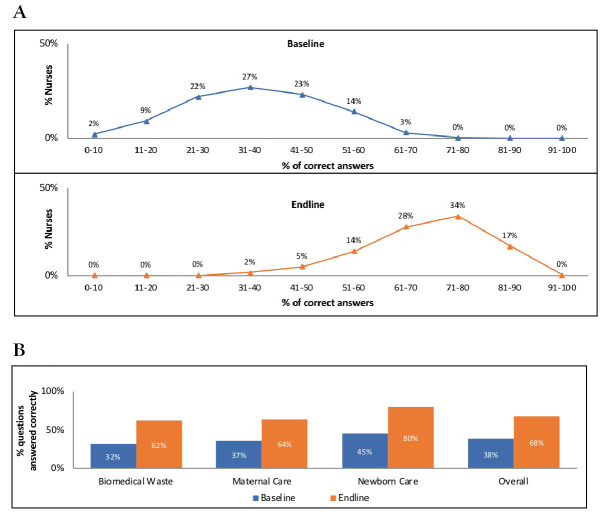
Nurse-mentee knowledge before and after the AMANAT intervention. **Panel A**. Changes in the proportion of nurses responding correctly to knowledge questions between baseline and endline. **Panel B**. Changes in proportion of questions correctly answered between baseline and endline by content. AMANAT – *Apatkalin Matritvaevam Navjat Tatparta*, translated emergency obstetrical and neonatal readiness. All shown differences between baseline and endline proportions are statistically significant at *P* < 0.001.

Nurse-mentees’ knowledge improved significantly after the intervention. Only 17% of nurse-mentees interviewed at baseline compared with 93% of those interviewed at endline answered >50% of questions correctly; none of them answered >70% of questions correctly at baseline, while 51% did so at endline (**Figure 2**, Panel A). On average, nurse-mentees answered correctly 38% of questions at baseline and 68% of questions at endline (*P* < 0.001), with knowledge of neonatal care aspects being relatively higher than knowledge of maternal intrapartum care or general biomedical waste management (**Figure 2**, Panel B).

### Infection control practices

**Figure 3:**
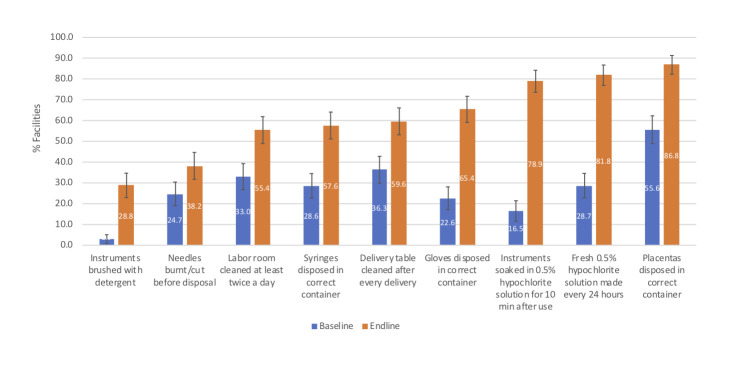
Infection control practices before and after the AMANAT intervention. AMANAT – *Apatkalin Matritvaevam Navjat Tatparta*, translated emergency obstetrical and neonatal readiness. Columns are proportions and corresponding 95% confidence intervals of facilities performing each of the infection control measure shown. All differences between baseline and endline are statistically significant at *P* < 0.006 or better.

All nine infection control measures assessed were significantly more prevalent at endline than baseline (all *P* < 0.006; **Figure 3**). Seven of these measures were practiced in >50% of facilities at endline, of which two (ie, daily preparation of 0.5% hypochlorite solution for soaking medical instruments and disposal of placenta in correct containers) were observed in >80% of facilities.

### Intrapartum and newborn practices

Performance of the 22 intrapartum and 13 newborn practices assessed varied widely at baseline (**Table 2**). We found statistically significant improvements in 18 intrapartum practices (all *P* < 0.05), all of which were performed with >50% of deliveries observed at endline. We also noted significant improvement in the uptake of nine newborn care practices (*P* < 0.05). Three of the four newborn practices for which we did not document a significant uptake at endline were already at >95% coverage at baseline (ie, nothing applied to the cord stump, newborn weighed, birth recorded in the register); the fourth was the match between observed and recorded birthweight, noted in 35.6% of deliveries observed at baseline and 42.6% of deliveries observed at endline. Also of note, the most important improvements in essential newborn care were found with practices performed in <35% of deliveries observed at baseline: cord checked for pulsations before clamping, sterile blade or scissors used to cut the cord, early initiation of skin-to-skin care, and use of sterile gauze for wiping newborn’s eyes. While the last practice mentioned was performed in only 15.8% of deliveries, all other practices were observed in 42.6%-99.8% deliveries at endline.

**Table 2:** Essential intrapartum and newborn management practices before and after the AMANAT intervention

Observed deliveries	Baseline	Endline
**Proportion (95% CI)**	
***Intrapartum management practices***	**N = 763**	**N = 628**
Women received the following at any point before delivery:		
-temperature check	2.9 (1.8-4.4)	35.4 (31.5-39.5)
-abdominal examination	8.4 (6.4-10.6)	66.6 (62.6-70.6)
-pulse measurement	17.1 (14.3-20.0)	79.8 (76.4-83.0)
-fetal heart rate measurement	16.9 (14.3-19.8)	84.8 (81.6-87.6)
-blood pressure measurement	45.5 (41.4-48.8)	86.9 (84.0-89.6)
-vaginal examination	93.7 (92.1-95.5)	93.5 (91.5-95.5)
Fundal pressure not applied anytime during labor	60.3 (56.4-63.8)	83.6 (80.6-86.6)
All delivery conductors used apron and mask	4.6 (3.2-6.4)	26.4 (22.8-30.4)
All delivery conductors washed hands	32.4 (28.8-35.1)	75.1 (71.1-79.0)
All delivery conductors wore gloves	89.1 (86.4-91.3)	92.9 (90.4-94.9)
Oxytocin (or combination of) given in proper dose by route within 1 min of delivery	23.2 (19.1-26.8)	72.2 (68.0-76.0)
Patient received controlled cord traction	18.4 (15.4-21.8)	74.0 (70.1-77.6)
Patient received uterine massage after delivery of placenta	49.8 (46.0-53.5)	85.6 (82.4-88.4)
Patient received local anesthesia before episiotomy	16.5 (5.5-34.6)	50.0 (26.0-74.0)
Sterile scissors used during episiotomy	6.4 (0.8-21.8)	50.1 (26.2-74.2)
Patient given local anesthesia before repair	50.2 (38.7-60.8)	65.4 (53.9-75.4)
Episiotomy/tear repair sutured	58.3 (48.2-68.6)	83.1 (73.4-90.2)
Placenta lobes & membranes checked for completeness	2.1 (1.2-3.5)	44.8 (40.2-48.2)
Genital tract explored after delivery	54.6 (50.4-57.8)	66.2 (61.4-69.8)
Perineum cleaned at the end of delivery	9.4 (7.2-11.6)	28.5 (24.8-32.2)
Deliveries where all instruments/equipment used were sterile or disinfected	4.4 (3.0-6.2)	62.2 (57.2-65.8)
Delivery summary noted in case paper	55.8 (52.6-61.2)	89.5 (86.5-91.9)
Newborn management practices*	**N = *749***	**N = *605***
Newborn placed on the mother abdomen immediately after birth	56.4 (52.6-60.6)	94.4 (91.9-95.9)
Newborn covered with a clean, dry cloth (separate from the drying cloth)	43.2 (39.4-47.0)	64.5 (60.5-68.5)
Cord checked for pulsations before clamping	6.8 (5.1-9.0)	67.4 (63.3-71.4)
Correct timing of cord clamping (>2 min after birth)	63.6 (59.6-66.8)	85.3 (81.6-88.2)
Cord tied with sterile clamp or thread	32.4 (27.2-37.8)	78.2 (74.6-81.6)
Sterile blade or scissors used to cut the cord	10.3 (8.4-12.8)	66.2 (61.2-7`.2)
Nothing applied to cord stump	98.8 (97.7-99.5)	99.8 (99.0-100.0)
Newborn eyes wiped with sterile wet gauze	4.4 (3.0-6.2)	15.8 (12.8-18.8)
Newborn weighed	98.5 (97.3-99.2)	97.8 (96.2-98.9)
Observed and recorded birthweight matched	35.6 (32.0-38.2)	42.6 (38.0-47.0)
Skin to skin care initiated within 5 minutes of birth	27.7 (24.2-31.0)	55.6 (51.4-60.3)
Breastfeeding initiated within 1 hours of birth	84.4 (80.9-87.4)	90.3 (87.3-93.2)
Birth recorded in birth or delivery register	95.6 (93.6-97.2)	97.8 (95.8-99.2)

AMANAT – *Apatkalin Matritvaevam Navjat Tatparta*, translated Emergency Maternal and Neonatal Care Preparedness, CI – confidence interval

*Newborn practices assessed when observed deliveries ended in live birth.

### Results from regression analyses

Between baseline and endline, nurse-mentees’ mean knowledge score increased by 84.6%, the mean facility infection control and maternal intrapartum management scores more than doubled, and the mean newborn management score increased by more than half (52.4%; all *P*-values <0.05; **Table 3**). Endline scores ranged between 56.8% and 72.8% of maximum scores for all outcomes. In regression analyses, considering the maximum possible scores through outcome score rescaling and after adjusting for potential confounders, we found that the AMANAT intervention led to significant increases in nurse-mentees’ knowledge (30.1%), facility infection control (30.8%), intrapartum (29.4%), and newborn management (24.2%) practices (all *P*-values <0.05). Following the AMANAT intervention, facilities of a higher level than PHC and those with higher equipment scores had better gains in nurse-mentees’ knowledge and facility infection control scores; nurse-mentees in facilities with higher equipment scores also had significantly higher intrapartum and neonatal management scores (all *P* < 0.05; data not shown).

**Table 3:** Associations between nurse-mentees’ knowledge and practices and receipt of the AMANAT intervention

Outcome	Baseline	Endline	*P*-value	Adjusted β coefficient for AMANAT intervention†,‡
**Mean score (SD), Cronbach’s alpha**	
Knowledge score* (30 items; score max = 30)	11.7 (4.3), 70.8%	21.6 (3.7), 70.6%	<0.001	30.1 (28.1-32.1), R^2^ = 0.57
Infection control score* (9 items; score max = 9)	2.4 (1.8), 60.3%	5.5 (2.1), 67.8%	<0.001	30.8 (25.6-36.0), R^2^ = 0.39
Intrapartum management score* (22 items; score max = 22)	5.9 (2.3), 58.4%	12.5 (3.4), 72.8%	<0.001	29.4 (26.6-32.2), R^2^ = 0.626
Newborn management score* (13 items; score max = 13)	6.3 (1.8), 51.6%	9.6 (2.1), 56.8%	<0.001	24.2 (20.8-27.6), R^2^ = 0.44

AMANAT – *Apatkalin Matritvaevam Navjat Tatparta*, translated Emergency Maternal and Neonatal Care Preparedness, SD – standard deviation

*For all scores, items assessed are scored as 1 if known correctly/performed and 0 if unknown/not performed; reported mean scores are not transformed.

**†**Scores were linearly transformed to a 0-100 scale for use in regression analyses; all models adjusted for type of facility, annual delivery volume, equipment score, AMANAT phase, and clustering at the nurse-mentor team level; the knowledge model is also adjusted for mentee’s experience (years); the intrapartum and newborn management models are also adjusted for delivered woman’s parity; the time when delivery observation started; time during day when taken to the labor room, and the highest provider qualification among providers assisting with the delivery.

‡Parameter estimates indicate the mean change out of 100 (ie, can be interpreted as percentage change) in summary practice score following the intervention. Positive parameter estimates indicate that the intervention is associated with improvement in knowledge/practices. All associations shown are statistically significant at *P*-level <0.05.

## DISCUSSION

The AMANAT intervention was an emergency response to a health workforce capacity crisis in the face of a rapid increase in the number of women delivering in public facilities. It was not aimed to function as a structural change or a replacement for in-service or other training methods for EmONC providers in Bihar. Rather, it was designed to overcome the pitfalls of other capacity building methods by being minimally disruptive to service delivery and by including a mentoring component that motivates providers and amplifies behavior and clinical practice changes. Offered as part of a comprehensive health system strengthening and quality improvement initiative in public facilities, this intervention also aimed to instill a sense of professional pride and shared interest among EmONC providers to offer quality services [6].

AMANAT led to increased adherence to evidence-based care and improved performance of essential intrapartum and newborn practices by nurses in public BEmONC facilities in Bihar. Exposure to the intervention improved nurse-mentees’ knowledge and performance of infection control and intrapartum practices by about 30%, and their performance of essential newborn practices, which was already higher at baseline, by 24%.

Among facility-level infection prevention measures, the appropriate cleaning and disinfection of delivery instruments saw the most impressive uptake between baseline and endline assessments. Also, provider-level practices to control infection before and during delivery (ie, hand washing; use of disinfected, sterile instruments) were considerably more prevalent after the intervention. Especially encouraging are changes observed in the initial assessment (ie, vital sign checking, fetal heart rate monitoring) of pregnant women upon arrival at the facility and before delivery – without a comprehensive initial examination, it is difficult to recognize or prevent potential complications. Importantly, with postpartum hemorrhage being a key contributor to maternal mortality in India [15], we found promising changes in the active management of third stage of labor practices known to reduce occurrence of severe postpartum hemorrhage by 60%-70% [16]. After the intervention, in about 72% of deliveries observed, oxytocin was given in the proper dose (10 IU) intramuscularly within 1 minute of delivery; controlled cord traction was practiced in 74% of deliveries observed; and uterine massage after the delivery of the placenta accompanied about 86% of deliveries observed. These represent changes of about 50%, 56%, and 366%, respectively, as compared to baseline. Areas of intrapartum care that were slower to change included use of personal protective equipment (ie, apron and masks) with all deliveries, episiotomies and related use of local anesthesia, temperature checking during initial assessment, checking of placenta for completeness, and cleaning the perineum after delivery. Also notable are the observed improvements in newborn cord care, placement on the mother’s abdomen immediately after birth, and early initiation of skin-to-skin care.

Our study is not without limitations. Given facility readiness differences, a cluster-randomised design evaluation was deemed infeasible. The pre-post design and the use of a uniform process of selecting facilities in each AMANAT phase minimised the effect of between-facility variations and allowed for facilities to be reached within the stipulated time. Baseline and endline data were not available for all outcomes in all facilities where AMANAT was implemented. However, available state-level data on key facility characteristics are reassuring with regard to the statewide representativeness of our results. Of the 495 public BEmONC facilities in Bihar, 83% are PHCs; they are served, on average, by 12 health workers and offer about 2200 deliveries each year. By comparison, among AMANAT facilities in our analysis, 78% were PHCs, they had an average of 11 health workers, and performed 2296 deliveries per year [14]. Only normal deliveries that occurred during the day were observed at both baseline and endline. This was because pregnancy complications were not frequent enough to be reliably observed in all facilities and in order to minimise the potential influence of complication severity and differences in 24/7 availability of human resources on our findings. Observations were only conducted Monday-Friday during daytime hours, which may have overestimated the effects of the intervention on essential maternal and newborn practice uptake given potential staff shortages that are more likely to occur during weekends and evenings.

There is limited documentation of experience with and impact of mentoring and training interventions on improving providers’ EmONC knowledge and skills in low- and middle-income countries [5,7,8,10-12,17-19]. For such skill building, they appear to be superior to traditional, short-duration trainings away from health facilities because they offer the option of both simulated and bedside learning and practicing [5], but their effectiveness varies based on context, barriers and facilitators to implementation [12]. Nonetheless, all studies to date furnished positive evidence regarding the acceptability of such interventions. Most recently, a qualitative study in Karnataka, India found that mentors provided training and support in a non-threatening manner, and mentees perceived them as helpful, trusted resources; nurse-mentees frequently contacted nurse-mentors between trainings to seek advice or confirm clinical decisions, which helped ingrain clinical skills and adherence to guidelines into daily practice [17]. Our results add to this body of evidence.

There are lessons to be learned from the AMANAT intervention in Bihar. First, there is value in having started with a sufficiently large, pilot program that provided information on feasibility, acceptability, design, and potential success of the intervention upon scale-up. This is demonstrated by the little variation in outcomes by implementation phase (data not shown). Second, the intervention could not have been implemented with fidelity at such large scale without the full collaboration of the GoB and of providers working in public facilities. Mentors’ ability to train and prompt nurse-mentees’ changes in knowledge and practices could have been completely undermined if medical officers or facility leadership were not in favour of the intervention. Third, the intervention was offered within a larger, statewide quality improvement framework in public facilities. As a result of this initiative, we found improvements in available and functional equipment in intervention facilities between baseline and endline assessments, and in regression analyses, the equipment score was found to be a significant positive predictor of nurse-mentees’ knowledge and practice scores. Fourth, joint mentoring by 2 nurse-mentors in each facility, despite its cost implication, allowed them to divide responsibilities and engage more intensively with the nursing staff. Busy facilities were expected to require more mentoring support than lower volume facilities [17]. Yet, we did not find that delivery volumes influenced mentees’ knowledge or performance in a significant manner. On the other hand, we found significantly higher scores of mentees’ knowledge and practice of general infection prevention measures in higher than PHC-level facilities. This may be due to having better trained nurses (ie, more GNMs) in these facilities or to having a higher proportion of complicated cases in these facilities and thus more opportunities for nurse-mentees to learn and practice. Fifth, some elements of the AMANAT intervention should be considered in the design of future training interventions for EmONC providers in Bihar. The mentoring model can assist with scalability and sustainability of future training interventions as mentees not only become agents of practice and quality culture changes in the facilities where they work, but potentially beyond if further trained to serve as nurse-mentors. This will also address the limitations with having to bring nurse-mentors from outside the state. Given the challenges with finding nurse-mentors in Bihar for training, the intervention was modified early in 2018 to no longer rely on availability of mentors from outside the state. Professional organisations worldwide recommend the use of simulations for in-service training in obstetrics and neonatology [20] – simulation should be incorporated in future EmONC provider trainings in Bihar if funding is available. Simulation not only gets the team involved but allows them to practice in a more realistic environment where there is no risk to actual patients. To build skills in recognising and managing pregnancy complications, mentees could be rotated through high-level facilities to observe and practice in different environments. Inclusion of team-based problem solving and accountability techniques in future training curriculum may lead to wider adherence to clinical guidelines than accomplished by AMANAT. Also, use of debrief meetings that potentially involve labor and delivery units’ leadership can help share progress and action plans for continuous quality improvement. Finally, despite positive results, AMANAT endline scores represented about 72%, 61%, 57%, and 74% of maximum knowledge, infection control, intrapartum, and neonatal practice scores, respectively. Considerable gaps in knowledge and practice remained after the AMANAT intervention in facilities exposed to the intervention. Emergency response interventions like AMANAT are resource-intensive, cannot fully solve broad health workforce capacity problems, and cannot replace either other training methods or the need for close oversight of the quality of intrapartum care. Quality improvement initiatives that include teamwork and communication as well as simulation trainings are expected to close the gaps in knowledge as well as the gap between knowledge and practice.

## CONCLUSION

Learning from the AMANAT intervention and building on a much stronger health system in Bihar in 2019 than 2011 [6], future mentoring and training interventions need to address current knowledge and practice gaps among EmONC providers and institutionalise a focus on quality of care and patient safety.
